# Advances in Early Breast Cancer Risk Profiling: From Histopathology to Molecular Technologies

**DOI:** 10.3390/cancers15225430

**Published:** 2023-11-15

**Authors:** Carlo Pescia, Elena Guerini-Rocco, Giuseppe Viale, Nicola Fusco

**Affiliations:** 1 Division of Pathology, European Institute of Oncology IRCCS, 20141 Milan, Italy; carlo.pescia@ieo.it (C.P.); elena.guerinirocco@ieo.it (E.G.-R.); giuseppe.viale@ieo.it (G.V.); 2 School of Pathology, University of Milan, 20141 Milan, Italy; 3 Department of Oncology and Hemato-Oncology, University of Milan, 20141 Milan, Italy

**Keywords:** breast cancer, early breast cancer, biomarkers, risk assessment, prognostication

## Abstract

**Simple Summary:**

Risk stratification for early breast cancer (BC) is extremely relevant for tailoring clinical decisions but challenging due to the absence of comprehensive guidelines. Traditional criteria like tumor size, lymph node involvement, histological type, grade, lymphovascular invasion, and immune cell infiltration serve as significant prognostic indicators. Alongside hormone receptor, HER2, and BRCA1/2 testing, molecular subtyping through gene expression profiling offers valuable insights for personalized clinical decisions. “Omics” technologies, applicable to tissue and liquid biopsy samples, have expanded risk evaluation capabilities, with limitations due to the lack of prospective data in early BC. This research overview paper highlights the need for standardized methodologies and integrated pathological models across multiple analytical dimensions for earlu BC risk stratification. The aim is to provide a practical guide for histopathologists and molecular pathologists involved in early BC profiling.

**Abstract:**

Early breast cancer (BC) is the definition applied to breast-confined tumors with or without limited involvement of locoregional lymph nodes. While risk stratification is essential for guiding clinical decisions, it can be a complex endeavor in these patients due to the absence of comprehensive guidelines. Histopathological analysis and biomarker assessment play a pivotal role in defining patient outcomes. Traditional histological criteria such as tumor size, lymph node involvement, histological type and grade, lymphovascular invasion, and immune cell infiltration are significant prognostic indicators. In addition to the hormone receptor, HER2, and—in specific scenarios—BRCA1/2 testing, molecular subtyping through gene expression profiling provides valuable insights to tailor clinical decision-making. The emergence of “omics” technologies, applicable to both tissue and liquid biopsy samples, has broadened our arsenal for evaluating the risk of early BC. However, a pressing need remains for standardized methodologies and integrated pathological models that encompass multiple analytical dimensions. In this study, we provide a detailed examination of the existing strategies for early BC risk stratification, intending to serve as a practical guide for histopathologists and molecular pathologists.

## 1. Introduction

Early breast cancer (BC) is defined by the presence of tumors confined to the breast, including tumors with an involvement of less than three locoregional lymph nodes [1]. This definition aligns with the TNM stages T1-3, N0-1, and M0, as established by the American Joint Committee on Cancer (AJCC) [2,3]. Despite the rarity of recurrences, effectively managing early BC poses a significant challenge: precise risk stratification [4]. Despite efforts in clinical studies to identify high-risk early BC, there is a lack of consistency in defining such cases. In the monarchE trial, high-risk early BC was defined as the presence of either four or more positive axillary lymph nodes or one to three positive axillary lymph nodes with at least one of the following features: grade 3 disease, a tumor size of 5 cm or larger, and a Ki-67 proliferative index of at least 20% [5]. A consensus paper by the IRIDE working group [6] attempted a definition of high-risk early BC to be applied to hormone-responsive cases, encompassing grade 3 histology, pT3-pT4 and/or pN2-pN3 staging, Ki67 > 30%, expression of estrogen receptors < 10% and/or progesterone receptors < 20%, high residual cancer burden after neoadjuvant therapy, and high-risk class based on gene profiling assays. However, while each of these factors has the potential to influence the overall risk of relapse, there is currently no available prediction tool that incorporates and assigns appropriate weights to each of them.

Accurately evaluating the risk of both early and late recurrence is extremely important for informing clinical management strategies [7,8]. In this respect, histopathological analysis, combined with biomarker testing, plays a pivotal role in improving patient outcomes [9,10,11,12,13,14,15]. Traditional histology criteria, including tumor size, lymph node involvement, histological grade, lymphovascular invasion, and immune cell infiltration, have long been acknowledged as significant prognostic indicators in early BC [14,16]. The evaluation of hormone receptor (HR) status, specifically estrogen receptor (ER) and progesterone receptor (PgR), along with HER2 status, constitutes another essential aspect of BC profiling [17,18,19]. Molecular subtyping via gene expression profiling offers supplementary insights beyond conventional histopathological criteria, aiding treatment decisions [20,21,22,23]. Another significant biomarker in a subset of early BC is represented by *BRCA1/2* mutational status, whose testing holds importance for risk stratification, patients’ selection for treatment with PARP inhibitors, and genetic screening in high-risk individuals [8].

Advancements in omics technologies, applicable to both tissue and liquid biopsy samples, have significantly broadened our tools for accurately assessing the risk associated with early BC [24]. Despite these advancements, several challenges persist for prognostication and therapy prediction in these patients. It is crucial to establish standardized methodologies and incorporate a multitude of factors into comprehensive pathological models. In this study, we provide an in-depth examination of the currently available biomarkers for early BC risk stratification. Each biomarker is thoroughly discussed, along with tailored testing strategies, aiming to offer a practical guide for histopathologists and molecular pathologists.

## 2. Dissecting the Pathology Report

### 2.1. Histopathology

Morphological analysis stands as the bedrock of BC clinical decision-making [25]. Consequently, histopathological parameters continue to provide invaluable insights into tumor characteristics and behavior, aiding in treatment determinations and risk stratification, albeit primarily offering prognostic insights. The conventional histopathological factors for early BC encompass tumor type, size, histological grade, lymph node involvement, and lymphovascular invasion. All these histopathological factors can be integrated into prognostic scoring systems, such as the Nottingham prognostic index (NPI), which integrates tumor size, grading, and lymph node status, as well as other online resources (e.g., predictPLUS and Adjuvant! Online) [26,27,28,29,30]. While traditional histopathological factors provide valuable prognostic information, they should be discussed in the context of a more comprehensive clinical and biological profiling.

#### 2.1.1. Tumor Type

Histologic type describes the specific morphological and architectural characteristics that define the microscopic appearance of BC [31]. Low-risk BCs encompass a range of tumor types, including low-grade non-special-type (NST) carcinomas, and special-type tumors such as tubular carcinoma, cribriform carcinoma, mucinous carcinoma, salivary-gland-type carcinomas, and tumors with apocrine differentiation [31,32,33]. These malignancies are characterized by a low frequency of genomic instability and often exhibit an indolent clinical course with a low rate of relapses [34]. Nonetheless, in cases like these, therapeutic approaches can be complex, especially when dealing with NST or special-type triple-negative breast cancer (TNBC).

#### 2.1.2. Tumor Size

The primary tumor’s largest dimension profoundly influences the prognosis, as larger tumor sizes are associated with a higher risk of metastasis and poorer outcomes [32]. The AJCC staging system incorporates tumor size (represented by the T specifier) to define BC stages. Considerations for determining the size of invasive carcinomas should be based on an assessment that combines macroscopic observations with microscopic examination and should be compared to clinical staging information [35,36,37]. According to the CAP cancer reporting protocol of invasive breast cancer (v4.9.0.0, available at https://www.cap.org/protocols-and-guidelines/cancer-reporting-tools/cancer-protocol-templates, accessed on 13 November 2023), for carcinomas larger than 1.0 mm but less than 1.5 mm, it is crucial not to round down to 1.0 mm; instead, round up to 2.0 mm. This precaution prevents the misclassification of tumors as pT1mi, which display survival rates very similar to those of ductal carcinoma in situ (DCIS)

#### 2.1.3. Histological Grade

All invasive breast carcinomas should undergo grading, specifically using the Nottingham combined histologic grade, which is an Elston–Ellis modification of the Scarff–Bloom–Richardson grading system [38,39]. High-grade tumors are associated with increased aggressiveness and poorer outcomes, while low-grade tumors tend to have a more favorable prognosis. The Nottingham combined histologic grade assesses three main factors: the extent of tubule formation, the degree of nuclear pleomorphism, and the mitotic count (or mitotic rate). This grading system should be employed for reporting purposes, as it establishes a correlation between the histologic grade and clinical outcomes within each stage grouping.

#### 2.1.4. Lymph Node Status

Lymph node involvement is another well-established prognostic factor in BC, determining, along with tumor size, the clinical and pathological stage of the disease (T and N specifiers) [40,41]. The presence of tumor cells within regional lymph nodes indicates a higher likelihood of systemic spread and is associated with a worse prognosis. The number of involved lymph nodes further refines the prognostic information, as well as the presence of extranodal extensions, which has been linked to inferior overall survival and disease-recurrence-free survival and should be incorporated into the pathology report [42]. The number of metastatic lymph nodes that define high-risk individuals in early BC can vary depending on the clinical guidelines and individual patient factors. However, commonly, the presence of four or more metastatic lymph nodes (pN2-N3) is considered a threshold for classifying a patient as high-risk in early BC [6], although such definition may be subject to changes and variations in both trials and clinical practice.

#### 2.1.5. Lymphovascular Invasion

Lymphovascular invasion (LVI) is defined by the presence of cancer cells within an endothelial-lined space outside the border of the invasive carcinoma, regardless of the vessel type (i.e., blood or lymphatics) [43]. The presence of LVI correlates with an increased risk of both lymph node involvement and metastatic dissemination, ultimately being associated with adverse outcomes, both in adjuvant and neoadjuvant settings [43,44,45,46,47,48,49,50,51], and should always be indicated in the pathology report.

### 2.2. Biomarkers

#### 2.2.1. Hormone Receptors

Assessing estrogen receptor (ER) and progesterone receptor (PgR) status using immunohistochemistry (IHC) is pivotal in BC management [52,53,54]. ER and PgR are key in hormonal signaling pathways, significantly influencing prognoses and treatment responses. ER-positive BCs (with >10% nuclear-positive neoplastic cells) constitute approximately 70% of cases, tied to milder disease progression and higher hormone sensitivity [55]. ER positivity implies tumor reliance on estrogen for growth and survival. Patients with ER-positive tumors have shown significant benefits from endocrine therapies [56]. PgR status is evaluated alongside ER status, as the expression of PgR is regulated via estrogen signaling [8,57]. Positive PgR status, defined as ≥1% neoplastic cells exhibiting nuclear staining, further refines the prognostic information and may have implications for endocrine therapy selection [58]. Tumors positive for both ER and PgR fall into the luminal group and generally have a better prognosis than dual-negative receptor tumors [59]. Importantly, the category of ER-low BC, introduced by 2020 ASCO/CAP guidelines and characterized by low ER expression (positivity in 1–10% of tumor cells), poses management and treatment challenges [60,61,62,63]. These tumors often exhibit aggressive characteristics, such as higher grade, larger size, and increased proliferation, and are associated with younger age, lymph node involvement, and poorer prognoses [62,64].

#### 2.2.2. Ki-67 Labeling Index

The Ki-67 index is a measure of cellular proliferation and is essential in the assessment of early BC risk [65,66]. High Ki-67 levels are associated with a more aggressive tumor phenotype and poorer prognosis [67]. Patients with a high Ki-67 index may have a higher risk of disease recurrence and benefit from more intensive treatment approaches, such as adjuvant chemotherapy [68]. Hence, the Ki-67 index helps in distinguishing between patients who may benefit from additional adjuvant therapy and those who could be spared from unnecessary interventions, although an internationally established final consensus on the Ki67 cut-off for therapeutic decisions has not been reached yet [69]. Nevertheless, a Ki67 20% cut-off is currently used to identify luminal B BC among HR+/HER2- cases, with obvious therapeutic implications [70,71,72]. According to the American Society of Clinical Oncology (ASCO) guidelines, in postmenopausal patients diagnosed with stage I-II BC, Ki67 expression might be used in combination with other clinical pathological factors to arrive at informed decisions regarding adjuvant endocrine and chemotherapy treatments when multigene assays are not accessible. Ki67 expression levels become particularly valuable for prognostic assessment when they fall below 5% (indicating low proliferation) or exceed 30% (indicating high proliferation), while distinguishing values within this range is essentially technically unreliable [73]. Interestingly, the American Food and Drug Administration (FDA) has recently approved abemaciclib, a CDK4/6 inhibitor, in combination with endocrine therapy in the adjuvant setting for HR+/HER2- and node-positive BC at high risk for recurrence with a Ki67 proliferative index of ≥20% (immunohistochemistry validated on Agilent platforms with the MIB1 clone). This approval stems from the results of the monarchE trial (NCT03155997), which, however, revealed a clinical benefit from the addition of abemaciclib to this subset of patients regardless of the proliferative status [5]. Consequently, Ki67 mainly serves as a prognostic factor for high-risk scenarios, rather than a response predictor [74]. It is important to acknowledge that using Ki67 as a predictive and prognostic marker is not devoid of debate [11]. Disputes involve antibody clone selection, scoring methods, reproducibility across labs, and the potential benefits of computer-assisted analysis or AI for Ki67 assessment. Efforts persist in establishing uniform guidelines and cut-offs for Ki67 assessment to enhance its consistency and reliability in clinical practice [70,75,76].

#### 2.2.3. HER2

Approximately 15–20% of BCs exhibit HER2 overexpression or amplification [77]. HER2-positive tumors tend to have a more aggressive clinical course, characterized by a higher risk of recurrence, and overall poorer prognosis [78]. However, targeted therapies like trastuzumab and pertuzumab have notably improved HER2-positive BC outcomes by inhibiting HER2 signaling pathways [79,80]. HER2 status is assessed using IHC for protein expression and reflex in situ hybridization (ISH) techniques for gene amplification. Standardized testing is used clinically to determine the eligibility for targeted treatments and guide decisions [81]. The integration of hormone receptor and HER2 testing into treatment algorithms has transformed the management of BC [32,82]. Additionally, HER2-low status, defined as HER2 FISH-negative cases with HER2 IHC 1+ or 2+ scores, has gained much interest lately due to the availability of new antibody–drug conjugates, such as trastuzumab–deruxtecan (T-Dx), which have been approved for previously treated metastatic BC [83,84]. Precise HER2 expression levels are vital, especially as the minimal level for T-DXd effectiveness is being studied. Given T-DXd’s potential benefits in HER2-zero (IHC 0) cases [85], the definition of HER2-low might change. Though HER2-low status currently does not impact early BC treatment, accurately defining HER2 status and reassessing it during progression and metastasis is essential for therapeutic strategies.

### 2.3. Tumor-Infiltrating Lymphocytes (TILs)

Tumor-infiltrating lymphocytes (TILs) have emerged as a critical component in the context of TNBC and HER2+ BC, where they hold a prognostic value [86]. According to the International TILs Working Group guidelines [87], TIL evaluation should encompass all mononuclear cells within the stromal/peritumoral compartment, expressed as a percentage relative to the stromal area itself. This evaluation should exclude intratumoral TILs, TILs outside the tumor border, TILs associated with intraductal carcinomas, as well as areas of necrosis or artifacts.

The abundance of so-called “stromal” TILs has been correlated to HER2+ and basal-like BC evaluated with gene expression profiling [88]. Moreover, higher TILs, with a threshold set at 20%, significantly predict pathological complete response (pCR) to neoadjuvant chemotherapy and better survival in HER2+ and TNBC [50,51,89,90,91,92]. Additionally, increased TILs have shown a correlation with improved outcomes following adjuvant therapy [93]. In detail, findings from the BIG-2-98 trial demonstrated that each 10% increase in TILs correlated with a reduced risk of relapse and death in node-positive TNBC, and conferred significant benefit in terms of disease-free survival in node-positive HER2+ patients treated with anthracycline-only chemotherapy [94]. Analyses from the FinHER trial revealed that TNBC with higher TILs at diagnosis experienced decreased distant recurrence rates, while HER2+ BC with higher TILs derived increased trastuzumab benefit [95]. Intriguingly, a 30% cut-off for TILs has been found to strongly up- and downstage traditional histopathological staging in BC, along with rendering histological grading irrelevant in terms of its prognostic implication in a pooled analysis comprising TILs; in light of these results, some authors have advocated for the integration of TILs in BC staging [96]. In the setting of early TNBC treated with adjuvant anthracycline-based chemotherapy with or without taxanes, a pooled data analysis [93] indicated a median TIL value of 23% at diagnosis; lower TIL levels were significantly associated with older age, larger tumor size, nodal involvement, and lower histological grade [97]. Notably, the quantity of TILs was significantly linked with improved survival outcomes in terms of OS, invasive-disease-free survival (i-DFS), and distant-disease-free survival (d-DFS), although TILs did not define the prognostic impact of different chemotherapy regimens. Moreover, node-negative TNBC with at least 30% TILs exhibited excellent survival outcomes, thus identifying 30% as an important exploratory cut-off in TIL evaluation and supporting its clinical validity in prognostication models [93]. A TIL cut-off of ≥30% has also demonstrated predictive value for excellent OS in stage I TNBC not treated with (neo)adjuvant chemotherapy [98]. The relationship between TILs and the response to PDL1-inhibitor-based immunotherapy has been variably explored as well [99,100], also in the neoadjuvant setting for TNBC [101,102,103,104,105]. Recognizing the significance of TIL scoring as a valid prognostic factor, the 2019 St Gallen Consensus [106] recommended its routine characterization and reporting, with 66% of the panelists endorsing this practice. However, 90% of the panelists were hesitant to base their treatment strategies, such as chemotherapy de-escalation, solely on TIL reports, as the clinical utility of TILs remains under scrutiny and not yet fully substantiated. Therefore, routine TIL reporting by pathologists may be suggested but not mandatory, as reiterated in the 2023 St Gallen Consensus [107]. Future developments, especially the integration of machine learning for enhanced TIL assessment, may potentially alter the current perspective [108,109].

## 3. Molecular Subtyping and Gene Expression Profiling

### 3.1. Gene Expression Profiling Assays to Inform Treatment

Gene expression profiling assays have revolutionized BC management by providing valuable prognostic and predictive information beyond traditional clinicopathological factors; they comprise the Oncotype Dx, MammaPrint, Prosigna, Endopredict, and BC index assays [48,110,111]. They enable personalized strategies, sparing patients from unnecessary chemotherapy treatments, as they aid in differentiating which patients could potentially gain advantages or disadvantages from the inclusion of chemotherapy alongside endocrine therapy, mostly in HR+/HER2- BCs [69]. Among different gene expression profiling assays, the Prosigna (PAM50) assay and 3.1.2 EndoPredict^®^ tests have, at present, been validated only at a prognostic level, while Oncotype Dx, MammaPrint, and BCI hold both a prognostic and predictive role. The ASCO guidelines (Table 1) [73] suggest the use of Oncotype Dx, MammaPrint, or BCI in postmenopausal or >50-year-old women with HR+/HER2- early BC, which is either node-negative or with 1–3 node metastases. In premenopausal patients, Oncotype Dx is recommended in node-negative HR+/HER2- patients, while in the case of 1–3 axillary lymph node metastases, chemotherapy is advised, regardless of the genomic assay results. Currently, information regarding genomic test applications in patients with ≥4 positive nodes is lacking [73].

Similarly, the European Society for Medical Oncology (ESMO) assigns level I of evidence (i.e., evidence derived from at least one large randomized controlled trial of good methodological quality or meta-analyses of well-conducted randomized trials without heterogeneity) and grade A of recommendation (i.e., strong evidence for efficacy with a substantial clinical benefit) for both Oncotype DX and MammaPrint, as shown in Table 2.

Of note, the ESMO and ASCO guidelines do not favor one test over another, but emphasize their value in complex scenarios where selecting optimal adjuvant therapy is uncertain, as in luminal B BCs with 1–3 positive axillary nodes [3].

Beyond the conventional luminal and non-luminal molecular subtypes, additional molecular subgroups have emerged. For instance, the claudin-low subtype is associated with stem-like features and immune system modulation, and it is defined by a poorer prognosis, a triple-negative phenotype, low genomic instability, mutational burden and proliferation levels, and high levels of immune and stromal cell infiltration [112,113,114]. Another example is the molecular apocrine subtype, which is characterized by androgen receptor expression, ER negativity, and frequent HER2 positivity, paired with aggressive clinical behavior [115]. These subtypes provide further insight into BC’s heterogeneity and could potentially shape future tailored therapeutic approaches.

### 3.2. BRCA1/2 Mutations

*BRCA1/2* mutations play a significant role in early BC development and management [116]. These mutations are autosomal-dominant inherited genetic alterations impairing the homologous recombination machinery that increases the risk of developing mainly breast and ovarian cancers. Detecting *BRCA1/2* mutations in early BC patients is essential for treatment choices and risk evaluation [3]. Patients with *BRCA1/2* mutations may have distinct tumor characteristics and treatment responses, as they are more likely to develop TNBCs, which are typically more aggressive and may require tailored approaches. *BRCA1/2* mutations also impact therapy sensitivity; for instance, these mutations heighten the effectiveness of platinum-based chemotherapy and PARP inhibitors, targeting DNA repair deficits with synthetic lethality effects [117]. Specifically, the OlympiA trial (NCT02032823) has demonstrated that in patients with HER2-negative early BC at high risk and harboring germline *BRCA1* or *BRCA2* mutations, the use of the adjuvant PARP inhibitor olaparib following local treatment and neoadjuvant or adjuvant chemotherapy resulted in significantly longer overall survival and survival without invasive or metastatic spread compared with the administration of a placebo [118,119]. Olaparib [120] and other PARP inhibitors, such as niraparib [121] or talazoparib [122], have demonstrated promising results also in the neoadjuvant setting for HER2-negative *BRCA*-mutated early BC, although no significant difference has been observed in terms of pCR between using PARP inhibitors alone or in combination with chemotherapy [123]. Therefore, identifying *BRCA1/2* mutations in early BC patients could guide treatment choices, potentially leading to improved outcomes [68]. Moreover, *BRCA1/2* mutations stretch beyond initial diagnosis and treatment, affecting ongoing management and risk assessment for both patients and their families. Carriers face higher risks of contralateral BC and ovarian cancer. To mitigate these risks, proactive measures like risk-reducing surgeries (e.g., bilateral mastectomy and bilateral salpingo-oophorectomy) and intensified monitoring are recommended. The identification of *BRCA* mutations in BC also triggers the consideration of genetic testing and surveillance for family members at risk [124,125]. *BRCA1/2* testing should be offered to high-risk groups, especially patients with strong familiarity for *BRCA*-related tumors, an early diagnosis (before the age of 50), a diagnosis of TNBC before the age of 60, male patients with BC, and/or patients with a personal history of ovarian cancer or second BC [3]. In essence, *BRCA1/2* mutations significantly impact early BC by shaping tumor traits, treatment responses, and long-term risk management. Detecting these mutations in patients informs treatments, facilitates evaluation of future risks, and guides interventions for patients and their high-risk family members.

## 4. Conclusions: Challenges and Future Directions

**Figure 1:**
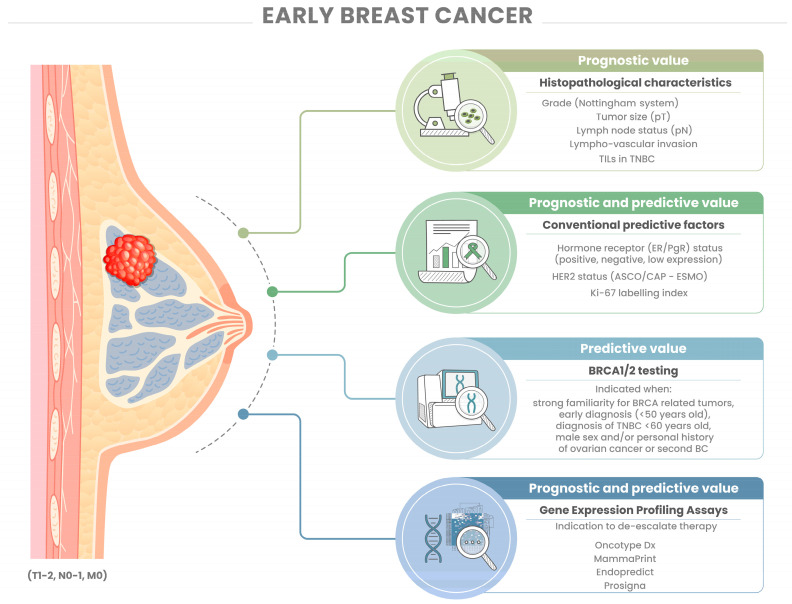
The risk assessment of early breast cancer (BC) involves analyzing various biomarkers and clinicopathologic features. Traditional histopathological characteristics like tumor size, lymph node involvement, histological grade, and lymphovascular invasion form the core of the pathology report, offering essential prognostic information. Some pathologists may report TILs, especially in TNBC and HER2+ BC, although current recommendations do not suggest basing therapeutic strategies on TILs due to their lack of clinical utility. Immunohistochemical assessment of hormone receptor status, HER2 status, and Ki67 is equally vital, reflecting the luminal/non-luminal molecular classification and guiding treatment choices with both prognostic and predictive implications. Integrating gene profiling assays into early BC evaluation optimizes adjuvant treatment decisions, especially for post-menopausal luminal patients with or without 1–3 node metastases and pre-menopausal luminal patients without lymph node involvement. In cases suggestive of hereditary BC syndrome, *BRCA1/2* testing is recommended. Abbreviations: BC, breast cancer; TILs, tumor-infiltrating lymphocytes; ER, estrogen receptor; PgR, progesterone receptor; TNBC, triple-negative BC.

Significant progress has been made in identifying prognostic and predictive factors for the treatment of early BC. Pathologists have played a fundamental role in BC biomarker definition and should be familiar with all the above-mentioned topics. However, challenges in biomarker testing and prognostic/predictive assays remain, and there are still opportunities for further research (Figure 1). One of the critical challenges is establishing standardized methods of assessing and interpreting biomarkers, including hormone receptors, HER2, Ki-67, TILs, as well as gene expression profiling assays. Consistency in methodologies and cut-off values is essential for reliable outcomes across various laboratories and clinical settings [68]. There are many unmet needs in the clinical management of early BC, ranging from the definition of high-risk disease to the optimal therapeutic choice in premenopausal node-positive patients, to the clinical utility of gene profiling assays in the choice of neoadjuvant treatment, to the need for the implementation of real-world data for proper biomarker validation. Gene expression profiling assays are recognized for their prognostic significance, yet their primary value lies in their potential or established predictive role in supporting chemotherapy de-escalation. Their use in early breast cancer risk assessment is currently not recommended. Further research is necessary to uncover novel biomarkers and molecular pathways that could enhance prognostic precision and explore new applications of existing tools. Many intriguing studies have explored the proteomic [126,127,128], metabolomic [129,130], and lipidomic [131,132] profiles of BC, with interesting insights that could be appliable to the early-stage disease. The integration of multi-omics data and machine-learning approaches could also provide insights into the intricate biology of BC and provide fruitful information in terms of risk assessment [133,134,135,136,137]. Collectively, these omics approaches contribute to broadening our comprehension of breast cancer, spanning from genetic susceptibility to molecular mechanisms. Nevertheless, their use in the present clinical practice is still a matter of controversy. Future clinical trials that embrace a comprehensive approach, integrating clinical, pathological, and molecular aspects, may significantly advance the risk assessment of early BC.

## Figures and Tables

**Table 1:** Recommended genomic tests in HR+/HER2- early breast cancer based on menopausal status, patient’s age, and number of lymph node metastases, according to the latest ASCO recommendations on gene profile assays, adapted from Andre F. et al. [73]. (***) high evidence quality and strong recommendation; (**) intermediate evidence quality and strong recommendation; (*) intermediate evidence quality and moderate recommendation.

N	Premenopausal or ≤50 Years Old	Postmenopausal or >50 Years Old
pN0	Oncotype Dx (***)	Oncotype Dx (***) MammaPrint (**) EndoPredict (*) Prosigna (*) BCI (*)
pN1a-c	Not recommended	Oncotype Dx (***) MammaPrint (**) EndoPredict (*) BCI (*)
pN2	Not recommended	Not recommended

**Table 2:** ESMO recommendations on gene profiling assays in HR+/HER2- early breast cancer, adapted from Cardoso et al. [3]. LoE, level of evidence; GoR, grade of recommendation.

Genomic Signatures	Method	LoE	GoR
Oncotype Dx	qRT-PCR	I	A
MammaPrint	DNA microarray	I	A
Prosigna	nCounter	I	B
EndoPredict	qRT-PCR	I	B
